# Computational modeling to study the impact of changes in Nav1.8 sodium channel on neuropathic pain

**DOI:** 10.3389/fncom.2024.1327986

**Published:** 2024-05-09

**Authors:** Peter Kan, Yong Fang Zhu, Junling Ma, Gurmit Singh

**Affiliations:** ^1^Department of Health Sciences, McMaster University, Hamilton, ON, Canada; ^2^Department of Health Sciences, Redeemer University, Hamilton, ON, Canada; ^3^Department of Mathematics and Statistics, University of Victoria, Victoria, BC, Canada; ^4^Department of Pathology and Molecular Medicine, McMaster University, Hamilton, ON, Canada; ^5^Michael G. DeGroote Institute for Pain Research and Care, McMaster University, Hamilton, ON, Canada

**Keywords:** computational modeling, DRG, Nav1.8, electrophysiology, neuropathy

## Abstract

**Objective:**

Nav1.8 expression is restricted to sensory neurons; it was hypothesized that aberrant expression and function of this channel at the site of injury contributed to pathological pain. However, the specific contributions of Nav1.8 to neuropathic pain are not as clear as its role in inflammatory pain. The aim of this study is to understand how Nav1.8 present in peripheral sensory neurons regulate neuronal excitability and induce various electrophysiological features on neuropathic pain.

**Methods:**

To study the effect of changes in sodium channel Nav1.8 kinetics, Hodgkin–Huxley type conductance-based models of spiking neurons were constructed using the NEURON v8.2 simulation software. We constructed a single-compartment model of neuronal soma that contained Nav1.8 channels with the ionic mechanisms adapted from some existing small DRG neuron models. We then validated and compared the model with our experimental data from *in vivo* recordings on soma of small dorsal root ganglion (DRG) sensory neurons in animal models of neuropathic pain (NEP).

**Results:**

We show that Nav1.8 is an important parameter for the generation and maintenance of abnormal neuronal electrogenesis and hyperexcitability. The typical increased excitability seen is dominated by a left shift in the steady state of activation of this channel and is further modulated by this channel’s maximum conductance and steady state of inactivation. Therefore, modified action potential shape, decreased threshold, and increased repetitive firing of sensory neurons in our neuropathic animal models may be orchestrated by these modulations on Nav1.8.

**Conclusion:**

Computational modeling is a novel strategy to understand the generation of chronic pain. In this study, we highlight that changes to the channel functions of Nav1.8 within the small DRG neuron may contribute to neuropathic pain.

## Introduction

Neuropathic pain is initiated or caused by a primary lesion or dysfunction in the nervous system (Hawksley, 2006; Finnerup et al., 2007) and it is a multidimensional condition that takes place along three different sites within the nervous system—peripheral, spinal, and supraspinal (Amir et al., 2005; DeLeo, 2006). It has become evident that peripheral neuropathic pain is characterized by membrane ectopic activity generated in both damaged as well as neighboring intact/surviving fibers of primary sensory neurons (Campbell and Meyer, 2006). These abnormal activities of peripheral neurons are suggested to play a role as a pain signal and as an inducer of central sensitization observed in animal models of peripheral neuropathy (Schaible, 2007).

Nav1.8 expression is restricted to sensory neurons. It produces the majority of the depolarizing inward current during an action potential (AP) (Blair and Bean, 2002) and has been reported to play an important role in a family of peripheral neuropathy (Faber et al., 2012) and other pathological pain animal models (Hameed, 2019). The biophysical characteristics of the Nav1.8 channel highlight its important contribution to repetitive firing and neuronal excitability. However, the specific contributions of Nav1.8 to neuropathic pain are not as clear as its role in inflammatory pain (Hameed, 2019). The high expression of Nav1.8 in nociceptors is reduced at both the mRNA and protein level in most, but not all, *in vivo* models of neuropathic pain (Boucher et al., 2000; Decosterd et al., 2002; Gold et al., 2003; Rogers et al., 2006), as well as in human patients (Coward et al., 2000). For example, axotomy or nerve transection, causes a downregulation of Nav1.8 expression (Dib-Hajj et al., 1996; Amir et al., 2005; Chen et al., 2011). Axotomy and spinal nerve ligation (SNL) reduces Nav1.8 expression by around 50%, while streptozotocin-induced diabetic neuropathy produces a 25% reduction (Okuse et al., 1997; Hong et al., 2004). Additionally, chronic constriction injury (CCI) elicits a decreased expression of Nav1.8 mRNA (Novakovic et al., 1998). Such reductions are thought to paradoxically conflict with the increase in ectopic firing that characterizes neuropathic pain. One proposed explanation involves a compensatory increase in tetrodotoxin (TTX) sensitive channels (i.e., Nav1.3) (Rogers et al., 2006; Hameed, 2019). Another hypothesis suggests that increased excitability in uninjured nociceptors could lead to an increase in the peripheral input, thereby contributing to the development of chronic neuropathic pain (Rogers et al., 2006; Hameed, 2019).

However, the regulation of Nav1.8 channel mechanisms in sensory neurons is complex (Chahine et al., 2005). In several models of neural injury, research has reported changes not only in the expression of the Nav1.8 channel but also in its voltage dependent kinetics (Gold et al., 1998, 2003; Moore et al., 2002; Black et al., 2004; Gold and Flake, 2005; Thakor et al., 2009). For example, it has been reported that the Nav1.8 current density was markedly decreased in injured dorsal root ganglion (DRG) neurons following CCI, while the voltage-dependent activation of the Nav1.8 channel in these neurons was shifted to depolarized potentials by 5.3 mV and inactivation shifted to hyperpolarized potentials by 10 mV (Li et al., 2015). On the other hand, it has been also reported that Nav1.8 mutation with small-fiber neuropathy shift activation in a 5.3 mV hyperpolarizing direction (Huang et al., 2013). Clarifying interactions between these changes in Nav1.8 in sensory neurons is an important step toward understanding the development of pathological pain.

Our previous investigation in a peripheral neuropathic animal model (NEP) also showed abnormal neuronal electrogenesis and heightened hyperexcitability in small DRG sensory neurons. The present study aims to understand how changes to the expression and kinetics of Nav1.8 modulate neuronal excitability, contributing to the various electrophysiological features observed in these neurons. We hypothesize that the increased excitability of DRG sensory neurons associated with neuropathic pain may be caused by changes in the kinetic properties of Nav1.8 channels as described above, which compensate for the effects of reduced Nav1.8 expression.

To test this hypothesis, we employed computer simulations utilizing a computational model of DRG neurons that feature Nav1.8 and other channels. Over the past few decades, a range of biophysical models representing different neural subpopulations have been developed, some of which are freely accessible from model databases such as ModelDB (Hines et al., 2004), NeuroML-DB (Birgiolas et al., 2023), and Open Source Brain (Gleeson et al., 2019). Simulations of the current models can describe the patterns of neural firing behavior by the relationship between the firing frequency and the injected current (Ma and Khadra, 2024). For example, Prescott et al. (2008) employed the computational models to describe the biophysical basis for three distinct dynamical mechanisms of action potential initiation. In this study, according to our data from acute intracellular electrophysiological recording, we constructed a single-compartment model of neuronal soma that contained Nav1.8 channels with the ionic mechanisms adapted from an existing small DRG neuron models from ModelDB (Mandge and Manchanda, 2018). Through computational modeling, we systematically analyzed the impact of interactions between channel parameters to gain a more complete understanding of Nav1.8 at the cellular level. We demonstrated that Nav1.8 is an important parameter in the generation and maintenance of abnormal neuronal electrogenesis and hyperexcitability. This suggests that Nav1.8 in an injured sensory neuron remains an important candidate responsible for neuropathic pain.

Computational modeling study is a novel strategy to understand the generation of chronic pain. The development of mathematical and computational modeling enabled us to incorporate the complex biological processes involved in pain perception, and helped us deepen our understanding of the role of the Nav1.8 sodium channel in neuropathic pain.

## Materials and methods

### Computational model

To study the effect of changes in sodium channel Nav1.8 kinetics, Hodgkin–Huxley type conductance-based models of spiking neurons were constructed using the NEURON v8.2 simulation software (Mandge and Manchanda, 2018). A single-compartment neuronal soma model that contained sodium currents and potassium currents was adapted from previous models in small DRG neurons (Baker, 2005; Mandge and Manchanda, 2018). Three forms of sodium current (TTX-s, Nav1.8 and Nav1.9), two types of potassium current [transient A-type (KA) channel (slowly-inactivating), and a delayed-rectifier (KDR)] were included in the simulation.

Briefly, the membrane potential was calculated using the following equation:

$$\frac{d\,V_{m}}{d\,t}=\frac{1}{C_{m}}\,\left(I_{s\,t\,i\,m}-I_{m\,e\,m\,b\,r\,a\,n\,e}\right)$$

I_Stim_ is the stimulus current, while I_membrane_ is the total ionic current contributed by cell membrane mechanisms such as pumps, exchangers, and channels.

I_membrane_ was described by the following equations:

I=membraneI+NaTTXSI+Nav1⁢.8I+Nav1⁢.9I+KAI+KDRIpas

*Where*, *I*_NaTTXS_ = *g*_NaTTXS_ *m*^3^_NaTTXS_ *h*_NaTTXS_ (*V*m – *E*Na);
*I*_Nav1.8_ = *g*_Nav1.8_ *m*^3^_Nav1.8_ *h*_Nav1.8_ (*V*m – *E*Na),
*I*_Nav1.9_ = *g*_Nav1.9_ * *m*_Nav1.9_ * *h*_Nav1.9_ (*V*m – *E*Na),
*I*_KA_ = g_KA_*n*_KA_(*V*m – *E*K).
*I_*KDR*_ = g_*KDR*_n 4_*KDR*_ (Vm – EK).*
*Ipas = gpas(Vm-Epas)*

(*V*m is the membrane potential, *E*Na, *E*K and Epas are the Na^+^, K^+^ and passive channels equilibrium potentials)

The equations used for calculating above m, n and h are:

$$\frac{d\,n}{d\,t}=\frac{n_{\infty }-n}{\tau_{n}}\,\frac{d\,m}{d\,t}=\frac{m_{\infty }-m}{\tau_{m}}\;\frac{d\,h}{d\,t}=\frac{h_{\infty }-h}{\tau_{h}}$$

m_∞_, h_∞_, and n_∞_ are the steady states of the activated sodium channel, inactivated sodium channel, and the potassium channel, respectively. m, h, n are the corresponding time constants.

#### Control computational model

For the control model, we adapted existing models of the small DRG sensory neuron [23] (ModelDB database, accession number: 243448). We simplified the model so that only the 5 main channels as described above are present. This strategy has been applied to other studies of small DRG sensory neurons in rats (Baker, 2005).

We then adjusted and validated the model against an experimental electrophysiological recording *in vivo* to test its robustness. This experimental recording was chosen to fit the average properties of experimental recording data including action potential shape and stimulation threshold.

Our control model neuron included morphological parameters based on available literature (Mandge and Manchanda, 2018): the soma was 24 μm in diameter, with a total membrane capacitance of 28 pF, Rm = 10,000 Ωcm^2^, Ra = 100 Ωcm. *T* = 25°C, Ek = −84.7 mV and ENa = 68.9 mV. These parameters resulted in a model with a somatic input resistance (Rin) of 553 MΩ, membrane capacitance (Cm) of 1.54 uF/cm. Based on our animal recording, we adjusted Erest from −53.5 mV to −62 mV, which was similar to the resting potential reported by other studies on DRG sensory neuron in rats (Du et al., 2014). Epas is calculated to −50 mV to achieve the Erest.

We also used individual channel equations from previous models (Mandge and Manchanda, 2018) to build our control model. Following, we used NEURON’s built-in “multiple run fitter” to achieve the best match between the experiment and the model. Since adjusted individual channel maximum conductance (gmax) are reported in other studies (Baker, 2005; Du et al., 2014) and may be due to the different expression of the channels between different species, age, sex and recording setting (Scheinman et al., 1989; Yang et al., 2019), therefore the fitter method was only limited to gmax of individual channels in this study. All other parameter values related to the individual channel kinetics in equations remained unchanged.

The gmax of individual channels was adjusted as follows:

gNaTTXS, from 0.0001 to 0.001 S/cm^2^
gNav1.8, from 0.0087177 to 0.0141 S/cm^2^
gKA, from 0.00136 to 0.00866 S/cm^2^
gKDR, from 0.002688 to 0.00388 S/cm^2^

#### Neuropathic computational model

**FIGURE 1:**
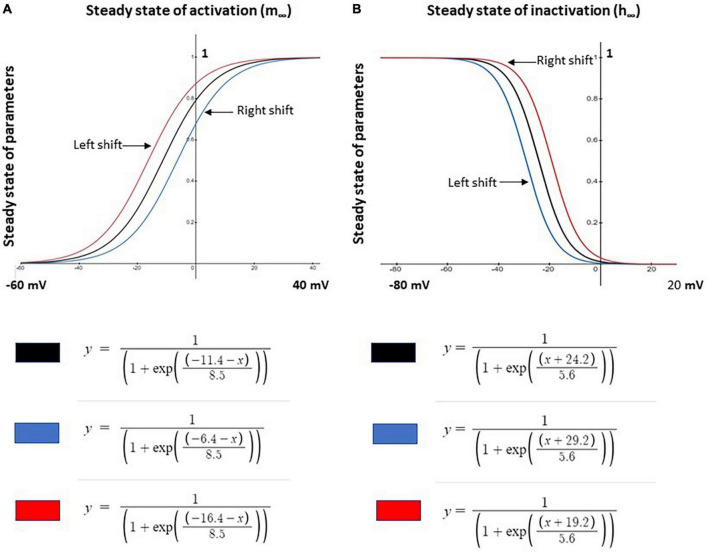
Shifts in steady state activation and inactivation values of Nav1.8 channel for neuron modeling. **(A)** Left –5 mV (red) and right +5 mV (blue) shift the voltage dependence of steady state activation (m∞) of the channel from the original (black) control model values. **(B)** Left +5 mV (blue) and right –5 mV (red) shift of the voltage dependence of steady state inactivation (h∞) of the channel from the original (black) control model values. Corresponding modeling equations are also shown.

Based on the control model, we simulated neuropathic models using parameter changes and values described in previous studies. The three Nav1.8 channel parameters varied in this study are maximal conductance (gmax), the steady state of activation (m∞), and the steady state of inactivation (h∞). These parameters were manually investigated individually or jointly. gmax was either increased or decreased ± 0.005 S/cm^2^ (+31.45%/−39.08%) while m∞ and h∞ were shifted by ± 5 mV and ± 5 mV, respectively, with corresponding equations shown in Figure 1.

### Animal model induction

All experimental procedures were in accordance with the Guide for the Care and Use of Laboratory Animals, Vols. 1 and 2, of the Canadian Council on Animal Care. All protocols were reviewed and approved by the McMaster University Animal Research Ethics Board.

#### Neuropathic animal model

Immunocompetent female Sprague-Dawley (SD) rats (Charles River Inc. St. Constant, QC, Canada) weighing 170–200 g randomly assigned to the NEP surgery group (*n* = 18). A peripheral neuropathy was induced according to the method previously described in detail (Mosconi and Kruger, 1996; Zhu and Henry, 2012).

Under anesthesia, the right sciatic nerve was exposed in the mid-thigh. Two 0.5 mm polyethylene (PE 90) tubing cuffs (Intramedic PE-90, Fisher Scientific Ltd., Whitby, ON, Canada) were inserted around the exposed nerve approximately 1 mm apart and the wound was then sutured.

#### Control animal model

In our previous study, control rats were induced using the same procedure except that no cuff was inserted around the sciatic nerve. It was shown that DRG neuronal membrane properties in sham rats were similar to unoperated control rats (Zhu et al., 2020). Therefore, in this study, we did not use a separate sham group.

### *In vivo* intracellular DRG recordings

Details of acute intracellular electrophysiological recording techniques have been reported in our previous studies (Zhu and Henry, 2012; Zhu et al., 2016, 2017, 2020, 2022). Briefly, each rat was anesthetized and fixed in a stereotaxic frame with the vertebral column rigidly clamped at lumbar L2 and L6. L4 DRGs containing large numbers of hind leg afferent somatic cells were selected and exposed for somata intracellular recordings. Sharp electrodes were used for *in vivo* recording. Excitability was measured by evoking action potentials (AP) in the soma of the DRG neurons using stimulation by direct injection of depolarizing current. To quantify soma excitability, with the aid of the “Protocol Editor” function in the pClamp 9.2 software program (Molecular Devices), the threshold of depolarizing current pulses injected into the soma was determined. This was achieved by applying current injections of 100 ms each, delivered with an amplitude of 0.5 to 2 nA with increments of 0.5 nA.

### Statistical analysis

We assessed and compared various parameters including AP amplitude and duration to describe AP shape, as well as threshold and number of spikes to evaluate neuron excitability between different simulations. Data is presented as mean ± the SEM and was analyzed with Mann–Whitney U tests for non-parametric data. All statistical tests and graphing were done using Prism4 software (Graphpad, La Jolla, CA, USA). The condition *P* < 0.05 was considered to indicate a statistically significant difference, as shown in the graphs.

## Results

### Comparison of neuropathic animal model and control animal model

**FIGURE 2:**
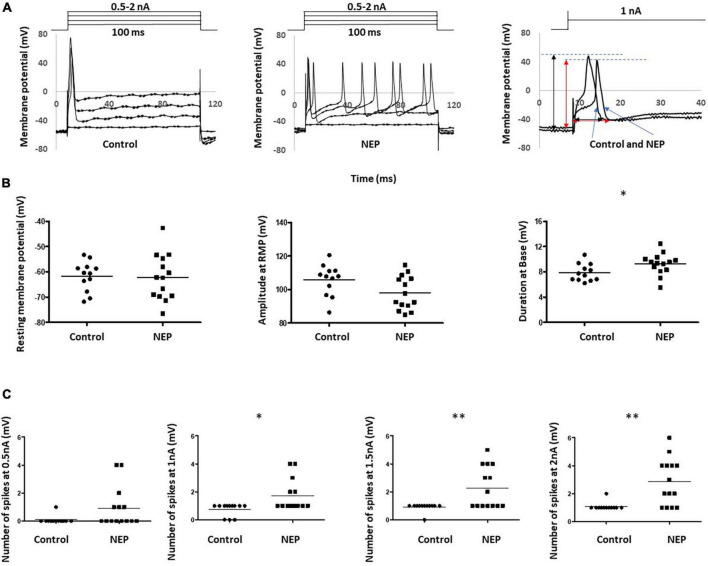
Abnormal neuronal electrogenesis and hyperexcitability of C-fiber sensory neuron in neuropathic animal model. **(A)** Examples of DRG neuron soma recording in control (left) and neuropathic rats (middle). Changes in Vm were generated by applying 100 ms rectangular current clamps of amplitudes ranging between 0.5 nA and 2 nA in steps of 0.5 nA. Recordings at 1 nA were separately shown (right). Amplitude and duration measurements were marked with a black line for the control rat and a red line for the neuropathic rat. **(B)** Comparison of action potential shape of DRG neuron soma recording in control and neuropathic animal models. a: Resting membrane potential, b: Amplitude from Rest membrane potential of AP, c: Duration at base of AP. **(C)** Comparison of excitability of sensory neurons. The number of spikes, corresponding stimulation at 0.5, 1, 1.5 and 2 nA with the rectangular current clamp for 100 ms. Scatter plots show the distribution of the variables with the median (horizontal line) superimposed in each case. Asterisks above the graph indicate the significant differences between control and neuropathic animals: *P < 0.05 **P < 0.01.

Intrasomal recordings *in vivo* were made from a total of 26 L4 dorsal root ganglion neurons. These included 12 and 14 C-fiber sensory neurons recording in 10 control rats and 10 neuropathic rats, respectively. Figure 2A showed two example recordings from control and neuropathic rats, respectively, by applying 100 ms rectangular current clamps of amplitudes ranging between 0.5 nA and 2 nA in steps of 0.5 nA.

The first evoked action potential in each neuron was used to determine any differences in configuration between control and neuropathic animals. The right panel of Figure 2A illustrated how the resting membrane potential, action potential amplitude (from resting membrane potential) and action potential duration (at base) were actually measured on the experiment models. We observed a trend of decreasing amplitude in neuropathic rats compared to control rats (105.7 ± 2.678 mV in control and 97.94 ± 2,268 mV in NEP, *p* = 0.0523). Furthermore, there were significant increases in AP duration in the neuropathic rat model (7.892 ± 0.398 ms in control, 9.246 ± 0.4553 ms in neuropathic, *p* = 0.0375). There is no significant difference in resting membrane potential between control and neuropathic model (−61.70 ± 1.704 mV in control and −62.11 ± 2.445 mV in NEP, *p* = 0.893) (Figure 2B).

There was a significant increase in excitability in the neuropathic rat model, with decreased spike threshold and increased number of spikes at 0.5–2 nA with 100 ms rectangular stimulation (Figure 2C).

### Validation of the control computational model

**FIGURE 3:**
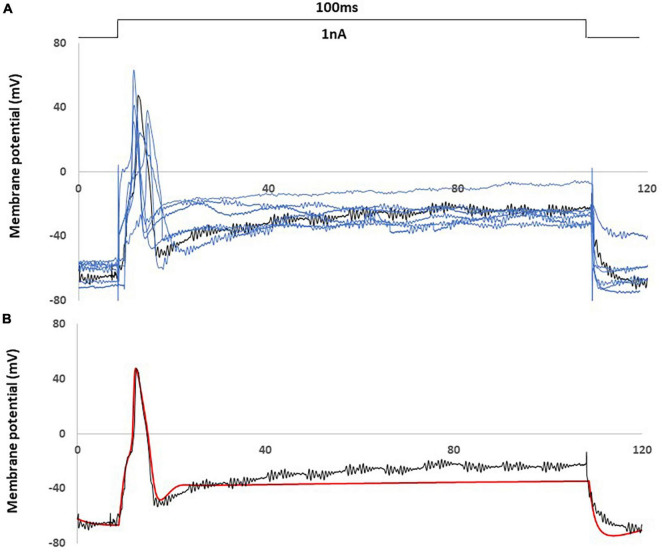
Computational simulation matches control animal recording data. **(A)** One experimental recording from the control rat (black) fit the average properties of control rat recording data (representative examples with blue) **(B)** Comparison of an action potential of small DRG neuron soma between this recording (black) and computational simulation (red). AP was generated by a current clamp of 1 nA, 100 ms rectangular current.

In Figure 3, a comparison was shown between an AP of the animal recording in control rat and an AP generated by the control computational model, with a current injection of 100 ms duration, 1 nA amplitude stimulation.

Figure 3A showed this particular animal recording closely captured the average AP shape (amplitude. duration) and the average behavior (resting membrane potential, spike threshold and single spike) among the recordings of control rats.

Figure 3B showed that the control computational simulation closely matched the AP properties of this specific animal recording. Table 1 shows the measured electrophysiological parameters, including resting membrane potential (RMP); action potential amplitude from resting membrane potential (APA); action potential duration at base (APD).

**TABLE 1:** Comparison of computational modeling and animal recording AP characteristics.

	Control animal	Control model	NEP animal	NEP model
Resting membrane potential	−63.5 mV	−62 mV	−59.8 mV	−62 mV
AP amplitude (APA)	111.21 mV	109.78 mV	106.68 mV	104.71 mV
AP duration at base (APD)	8.61 ms	8.56 ms	10.38 ms	11.01 ms

### Single parameter changes of Nav1.8 in computational model

The effects of single parameter changes of Nav1.8 were tested through individual changes of ± 0.005 S/cm2 to maximal conductance (gmax), ± 5 mV to the steady state of activation (m∞), and ± 5 mV to the steady state of inactivation (h∞) which is described in Figure 1.

**FIGURE 4:**
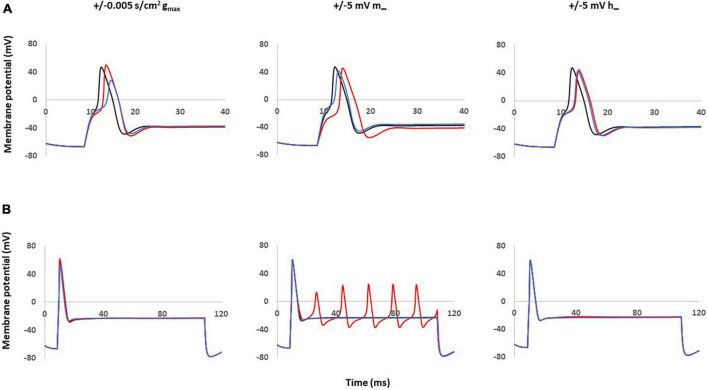
Single parameter changes of Nav1.8 channel effect on AP shape and excitability. Single parameter changes of Nav1.8 channel is described in the methods. Left panel: Comparison of control gmax (black) with decreased gmax (blue) and increased gmax (red). Middle panel: Comparison of control m∞ (black) with right +5 mV shift (blue) and left –5 mV shift (red) of m∞. Right panel: Comparisons of control h∞ with left +5 mV shift (blue) and right –5 mV shift (red) of h∞. gmax: maximum conductance; m∞: voltage dependence of steady state activation; h∞: voltage dependence of steady state inactivation. **(A)** Effect of Nav1.8 Changes on AP shape. AP was stimulated at threshold. **(B)** Effect of Nav1.8 Changes on AP excitability. APs generated with 2 nA, 100 ms stimulation.

Figure 4A shows the effects of single parameter changes of the Nav1.8 channel on AP shape at threshold stimulation. Changes in maximal conductance (gmax) altered the amplitude of the AP, while both left and right shifts in the steady state of activation and inactivation led to longer duration of AP. Figure 4B shows the effects of parameter changes on excitability.

Notably, only left −5 mV shift of the steady state of activation in Nav1.8 led to multiple spikes in stimulation ranges at 2 nA. The other parameter changes did not lead to the appearance of multiple spikes. The detailed comparisons of amplitude, duration, threshold and number of spikes at 0.5–2 nA are shown in Table 2 (part 1).

**TABLE 2:** Comparison of Nav1.8 changes in computational modeling with AP characteristics.

	AP amplitude (mV)	AP duration (ms)	AP threshold (nA)	Spikes at 0.5 nA	Spikes at 1 nA	Spikes at 1.5 nA	Spikes at 2 nA
**Single parameter changes of Nav1.8 in computational model**							
Control model	108.91	8.56	0.98	0	1	1	1
−0.005 s/cm^2^ G_max_	85.52	9.94	0.98	0	1	1	1
+0.005 s/cm^2^ G_max_	110.42	10.94	0.89	0	1	1	1
+5 mV m_∞_	102.886	8.48	1.12	0	0	1	1
−5 mV m_∞_	107.185	11.08	0.72	0	1	3	5
+5 mV h_∞_	110.6394	10.88	0.93	0	1	1	1
−5 mV h_∞_	105.056	10.86	0.92	0	1	1	1
**Coupled parameter changes of Nav1.8 in computational model**							
−5 mV m_∞_ and −0.005 s/cm^2^ G_max_	62.35	10.92	0.78	0	1	1	4
−5 mV m_∞_ and +0.005 s/cm^2^ G_max_	110.78	11.34	0.68	0	1	5	7
−5 mV m_∞_ and +5 mV h_∞_	106.81	12.50	0.72	0	1	1	1
−5 mV m_∞_ and −5 mV h_∞_	103.06	11.64	0.71	0	1	4	7
−5 mV m_∞_ and −5 mV h_∞_ and +0.005 s/cm^2^ G_max_	111.09	13.60	0.78	0	1	1	6
−5 mV m_∞_ and −5 mV h_∞_ and −0.005 s/cm^2^ G_max_	95.05	10.96	0.67	0	1	5	8

According to animal data analysis in Figure 2, the expected values of NEP computational model comparing control computational model should be decreased amplitude, increased duration, decreased spike threshold and multiple spikes at 2 nA. Red color marked AP characteristics of those computational models that match observed NEP animal recording. Blue color marked AP characteristics of those computational models that incapably match the expected values on NEP animal recording.

**FIGURE 5:**
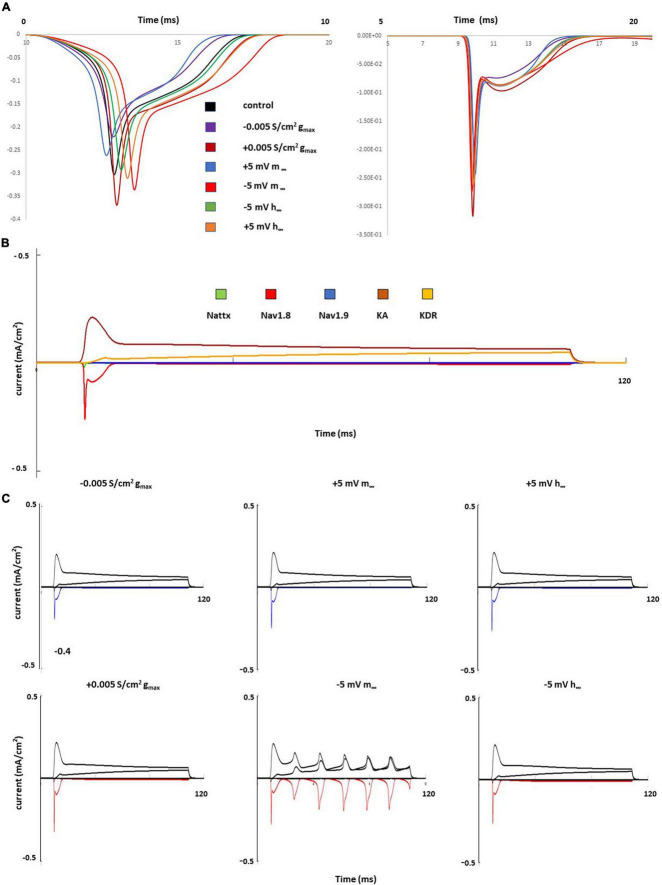
Single parameter changes of Nav1.8 channel effect on current. **(A)** Effect of Nav1.8 parameter changes on Nav1.8 channel current, at threshold (left) and 2 nA (right), each change was marked with a different color. **(B)** 2 nA, 100 ms stimulation on control model. Each channel current was shown with a different color. **(C)** Effect of Nav1.8 parameter changes on Nav1.8 (marked with blue or red) and other individual channel current, with 2 nA, 100 ms stimulation. Left panel: with decreased gmax (blue) and increased gmax (red); middle panel: right +5 mV shift (blue) and left –5 mV shift (red) of m∞; right panel: left +5 mV shift (blue) and right –5 mV shift (red) of h∞.

Figure 5 shows individual channel current with and the effects of single parameter changes of the Nav1.8 channel on each individual channel currents. The left shift in the steady state of activation leads to larger and broader inward currents of the Nav1.8 channel, both in stimulation at threshold and 2 nA (Figure 5A). Comparing the control model (Figure 5B), the currents in other channels also showed corresponding changes with the Nav1.8 channel (Figure 5C).

### Coupled parameter changes of Nav1.8

Based on the single parameter changes, we found that the left shift in steady state of activation played an important role in the AP shape and the excitability; however, this change did not produce a similar AP amplitude shape (decreased amplitude) observed in our neuropathic animal models. We then constructed computational models combining this change coupled with changes to the maximum conductance and the steady state of inactivation.

**FIGURE 6:**
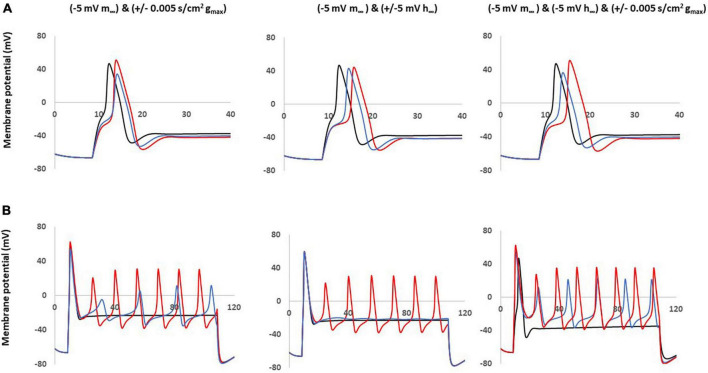
Coupled parameter changes of Nav1.8 channel effect on AP shape and excitability. Coupled changes combining left –5 mv shift of m∞ which described in Figure 4 with the other parameters. Left panel: with decreased gmax (blue) and increased gmax (red). Middle panel: with left +5 mV shift of h∞ (blue) and right –5 mV shift of h∞ (red). Right panel: with right –5 mV shift of h∞ and decreased gmax (blue); with right –5 mV shift of h∞ and increased gmax (red); gmax: maximum conductance; m∞: voltage dependence of steady state activation; h∞: voltage dependence of steady state activation. **(A)** Effect of Nav1.8 Changes on AP shape, which is stimulated at threshold, 100 ms. **(B)** Effect of Nav1.8 changes on AP excitability. APs generated with 2 nA, 100 ms stimulation.

These coupled parameter changes modulated the AP shape (Figure 6A) and excitability (Figure 6B). Notably, coupling a left −5 mV shift of steady state activation to a decrease in maximum conductance and/or to a right −5 mV shift of steady state inactivation reduced the AP amplitude and kept the broader duration. These coupled changes also maintained the decreased threshold and multiple spikes characteristic in neuropathic models.

The other coupled changes are incapable of matching all expected values at the same time. For example, coupling a left −5 mV shift of steady state activation to an increase in maximum conductance increased amplitude; coupling a left −5 mV shift of steady state activation with a left + 5 mV shift of steady state inactivation diminished the multiple spikes; and triple coupling a left −5 mV shift of steady state activation with a right −5 mV shift of steady state inactivation and an increase in maximum conductance still increase the amplitude.

The detailed comparisons of amplitude, duration, threshold and number of spikes at 0.5–2 nA and 100 ms stimulation are shown in Table 2 (part 2).

### Assessment of neuropathic computational models

**FIGURE 7:**
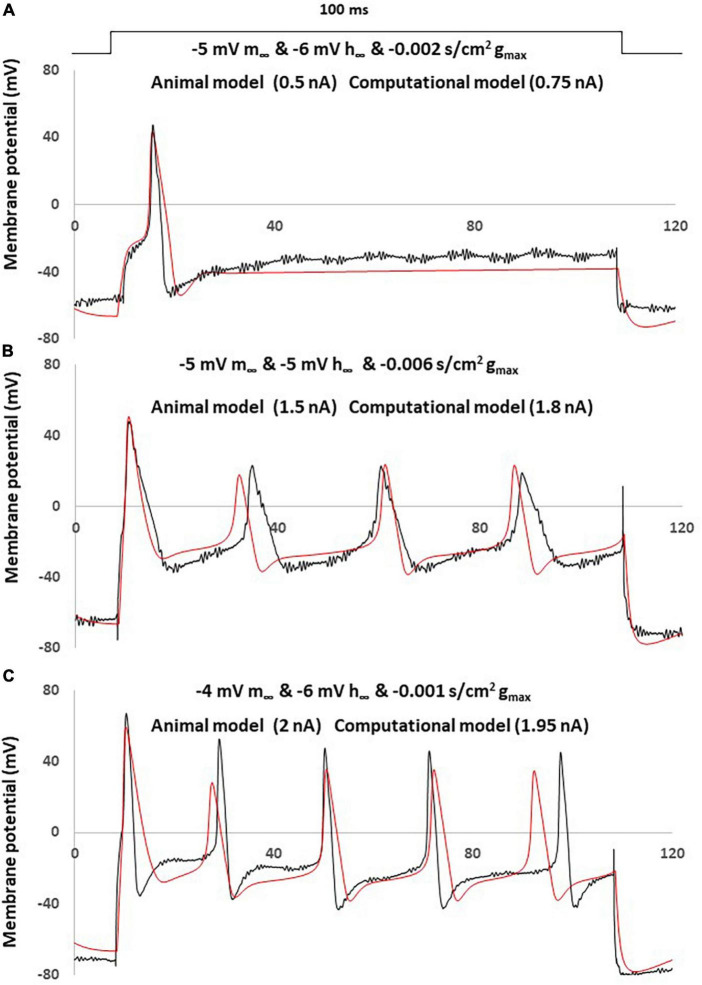
Simple computational simulation matches neuropathic animal recording data. Comparison of an AP between animal recording (black) and computational simulation (red). **(A)** Corresponding computational modeling was coupling changes with a left –5 mV shift of m∞, a right –6 mV shift of h∞ and decreased –0.001 S/cm2 of gmax. AP generated by a current clamp of 0.5 nA (animal recording) and 0.75 nA (computational modeling), 100 ms rectangular current. **(B)** Corresponding computational modeling was coupling changes with a left –5 mV shift of m∞, right –5 mV shift of h∞ and decreased –0.006 S/cm2 of gmax. AP generated by a current clamp of 1.5 nA (animal recording) and 1.8 nA (computational modeling), 100 ms rectangular current. **(C)** Corresponding computational modeling was coupling changes with a left –4 mV shift of m∞, right –6 mV shift of h∞ and decreased –0.001 S/cm2 of gmax. AP generated by a current clamp of 2 nA (animal recording) and 1.95 nA (computational modeling), 100 ms rectangular current.

The comparison between the NEP computational simulations and three different sensory neurons recording in neuropathic rats was shown in Figure 7.

Three computational models were built by coupling changes of a left shift of m∞, right shift of h∞ and decreased of gmax, to similar degrees with the above discussed changes. The other parameters were kept with no change.

The responses obtained in our neuropathic computational model are comparable to the neuropathic animal model, bearing close resemblance in terms of AP amplitude, duration and spike threshold (Figure 7A). The measured electrophysiological parameters, including resting membrane potential (RMP); action potential amplitude from resting membrane potential (APA); action potential duration at base (APD) was shown in Table 1. The other two examples also demonstrated similar patterns of multiple spikes with similar levels of rectangle 100 ms current stimulation (Figures 7B, C).

## Discussion

The specific contribution of Nav1.8 to neuropathic pain has been debated in previous studies, mainly due to a paradoxical reduction in expression which reduces inward current, yet still presents with increased excitability (Rogers et al., 2006; Hameed, 2019). However, potential shifts in steady state of activation and inactivation of Nav1.8 were reported in other peripheral NEP animal models (Li et al., 2015). Therefore, it is important to fully elucidate the interaction between changes to kinetic parameters and the expression of this sodium channel isoform in the induction and maintenance of DRG increased excitability in pathological pain states.

Although recently, development of Nav1.8-selective blocker, such as A-803476 and A-887826, which demonstrate the role of Nav1.8 in pain mechanism regarding its ability to modulate pain sensations, their usefulness as a research tool has been limited by these and other factors (Theile and Cummins, 2011). Additionally, isoform-specific sodium channel kinetic-altering pharmaceutical drugs have yet to be developed. The aim of this study was to deepen our current understanding of the likely roles of Nav1.8 in neuropathic pain using computational modeling. We studied the interactions of parameters using a computational model of a DRG neuron to reveal the mechanisms underlying the contributions of the Nav1.8 sodium channel in neuropathic pain.

Although the interaction of Nav1.8 sodium channel with other channels could vary depending on the types of other channels, we constructed a simplified model in which only the predominating channels are present, so that any differences in the firing of APs between the modified and control model could be attributed exclusively to changes in the Nav1.8 sodium channel conductance.

Our study revealed how Nav1.8 channels present in peripheral sensory neurons regulate neuronal excitability and induce various electrophysiological features on neuropathic pain. We observed abnormal neuronal electrogenesis and hyperexcitability in small DRG sensory neurons in our NEP animal model. Based on our computational simulation, we found that a left shift in the steady state of activation of the Nav1.8 sodium channel changed the AP shape, decreased the stimulation threshold and increased the number of spikes. Meanwhile, changes in maximum conductance and the steady state of inactivation alone did not result in multiple spikings, but played an important role when coupled to a left shift in steady state of activation by further modulating excitability and AP shape.

Based on our current results, the change of excitability observed in sensory neurons in neuropathic pain may not be due solely to a change in maximum conductance as most previous studies suggest (Rogers et al., 2006; Hameed, 2019), but can be dominated by a left shift (hyperpolarized) in the steady state of activation of Nav1.8. Our results are consistent with the previous studies of Nav1.8’s role in small-fiber neuropathy (Huang et al., 2013) and neuron hyperexcitability (Ye et al., 2015), where a hyperpolarized shift of activation of Nav1.8 increased excitability of sensory neurons. Notably, our data showed the opposite finding of the of CCI model where a depolarized shift of activation and hyperpolarized shift of inactivation of Nav1.8 increase excitability of sensory neurons (Li et al., 2015). It is not clear whether the difference between our animal model and the CCI model is related to an alternative mechanism of neuropathy. For example, the changes in Nav1.8 which lead to enhanced excitability might be related to their specific physiological range of membrane potential and the interaction of other channels.

Our computational model suggests that specific abnormal neuronal electrogenesis (wider duration, decreased amplitude of AP) and hyperexcitability (multiple spiking and decreased stimulation threshold) observed in our animal model can be orchestrated by coupled changes in Nav1.8, namely, a left shift in the steady state of activation, decreased maximum conductance and/or right shift in the steady state of inactivation. This result implies that the effect of reduced expression of Nav1.8 can be compensated by the left shift of steady state of activation in the NEP animal model. Our study adds to the body of evidence that Nav1.8 plays an important role in neuropathic pain with its abnormal expression and altered voltage dependent kinetics.

The limitation of this study is that we did not consider the correlation between Nav1.7 and neuropathic pain. Looking into current literature, the role of Nav1.7 in neuropathic pain is still unclear. It thus remains questionable whether Nav1.7 does contribute to the development of neuropathic pain (Hameed, 2019). However, the biophysical properties and high expression of Nav1.7 and Nav1.8 channels in nociceptors indicate both channels may play critical roles in determining the excitability of nociceptors, emphasizing their importance in normal pain-signaling. In future studies, specific isolated Nav1.7 channels instead of overall TTXS channels in neuropathic pain should be investigated.

Due to limited optimization of the Nav1.8 parameters, the comparison between computational and experimental models in this study is only qualitative and does not appear to be particularly quantitatively tight, especially in the neuropathic model. The other limitation of this study is that we did not add or subtract the dynamic Nav1.8 current to stimulate real sensory neurons. This is due to our animal recording data only using rectangular current clamps within general electrophysiological settings. Directly testing the role of particular channels in real neurons can be applied using dynamic clamps. In this configuration, a specific non-predetermined membrane current can be added to or removed from the cell while it is in free-running current clamp mode (Prinz et al., 2004; Berecki et al., 2014). This current is usually computed in real time, based on the recorded AP of the cell, and injected into the target cell. In future studies, investigators can inject simulated currents based on computational modeling results which describe the detailed channel-specific kinetic changes.

In conclusion, the role of Nav1.8 in the generation and maintenance of abnormal neuronal electrogenesis and hyperexcitability highlights the importance of this channel in the development of pathological pain. Our study provides a more complete understanding of this unique contribution to pain state at the cellular level that may allow for future developments of mechanism-based treatments for pain.

## Data availability statement

The raw data supporting the conclusions of this article will be made available by the authors, without undue reservation.

## Ethics statement

All experimental procedures were in accordance with the Guide for the Care and Use of Laboratory Animals, Vols. 1 and 2, of the Canadian Council on Animal Care. All protocols were reviewed and approved by the McMaster University Animal Research Ethics Board. The study was conducted in accordance with the local legislation and institutional requirements.

## Author contributions

PK: Data curation, Investigation, Methodology, Writing – review & editing. YZ: Data curation, Investigation, Methodology, Validation, Writing – original draft. JM: Data curation, Formal analysis, Methodology, Software, Writing – review & editing. GS: Conceptualization, Funding acquisition, Supervision, Visualization, Writing – review & editing.
